# Incidence of endophthalmitis after 23-gauge pars plana vitrectomy

**DOI:** 10.1186/s12886-018-0678-5

**Published:** 2018-01-23

**Authors:** Zhong Lin, Xiaofen Feng, Liya Zheng, Nived Moonasar, Lijun Shen, Ronghan Wu, Feng Chen

**Affiliations:** 1 The Eye Hospital, School of Ophthalmology and Optometry, Wenzhou Medical University, No. 270 West College Road, Wenzhou, Zhejiang, 325027 China; 2 Department of Surgery, Ophthalmology Unit, Faculty of Medical Sciences, University of the West Indies, St. Augustine, Trinidad and Tobago

**Keywords:** endophthalmitis, 23-gauge, pars plana vitrectomy, postoperative complication

## Abstract

**Background:**

Endophthalmitis is a rare but severe complication following PPV. The incidence of endophthalmitis varies between 20-gauge, 23-gauge, and 25-gauge incisions. The incidence and clinical features of endophthalmitis after 23-gauge PPV in an eye hospital in China was reported in this study.

**Methods:**

Data of the eyes that underwent 23-gauge PPV from January 2011 to December 2014 at the Eye Hospital of Wenzhou Medical University was retrospectively collected. All the information was obtained from the electronic medical system. The exclusion criteria included: (1) preoperative diagnosis of endophthalmitis; (2) history of vitrectomy; (3) intraocular surgery within 6 months; (4) history of ocular penetrating trauma; (5) sutures for any of the 3 sclerotomy incisions; (6) patients with cancer, acquired immune deficiency syndrome, or taking drugs that may influence the immune system. The diagnosis of endophthalmitis was based on clinical characteristics and/or culture results from an operative sample.

**Results:**

Three thousand nine hundred seventy nine eyes that underwent 23-gauge PPV surgery were included in this study. Among these eyes, 3 eyes developed endophthalmitis after surgery, giving an incidence of 0.075% (3/3979). The period in which endophthalmitis developed ranged from 1 to 5 days post-operation. The visual acuity decreased to hand motions or light perception postoperatively. The culture of aqueous and vitreous of the 2 eyes revealed *Staphylococcus epidermidis* and *enterococcus faecalis* respectively, however was negative for the third eye. All 3 eyes had a favorable response to the treatment of vitreous tap and intravitreal antibiotics injection. Two eyes gained visual acuity of 0.05 and 0.5, respectively at the final visit.

**Conclusions:**

Endophthalmitis is a rare but sight-threatening complication after 23-gauge pars plana vitrectomy. The peak duration of onset was within 5 days post-operation, with gram positive cocci being the common pathogenic organism.

**Electronic supplementary material:**

The online version of this article (10.1186/s12886-018-0678-5) contains supplementary material, which is available to authorized users.

## Background

Although rare, endophthalmitis is a vision-threatening complication following pars plana vitrectomy (PPV). The incidence of endophthalmitis after 20-gauge PPV was reported to be between 0.03 and 0.15% [1–4]. However, Scott et al. reported a significantly higher incidence of endophthalmitis after 25-gauge PPV (0.84%) during 2005-2006, compared to 20-gauge PPV (0.03%) [5]. Similar results were reported by Kunimoto et al. (20-gauge vs. 25-gauge: 0.018% vs. 0.23%) [6]. The authors speculated that unsutured sclerotomy wounds in small gauge vitrectomy may one of the important risk factors for the higher incidence of endophthalmitis [5, 6]. However, some studies reported the incidence of endophthalmitis after 25-gauge (0.03%) or 23-gauge (no endophthalmitis case) vitrectomy was not higher than that of 20-gauge vitrectomy (0.03%) [7, 8]. Scott et al. also reported stable rates of endophthalmitis after 20-gauge (0.02%) and 23 gauge (0.03%) PPV from 2007 to 2008, but a decreased rate after 25-gauge (0.13%) PPV, compared to 2005-2006 [9]. The authors speculated this decrease may related to the increase of surgeons’ experience [9]. A 5-year multi-center retrospective data from Latin America showed that the incidence of endophthalmitis did not vary much after small-gauge PPV, compared to 20-gauge PPV (all no more than 0.03%) [10]. Data which was gathered from a large prospective, nationwide case-control study suggested that the small-gauge vitrectomy did not increase the risk of endophthalmitis [11].

Reports of endophthalmitis after 23-guage PPV are relatively rare. The purpose of this study is to report on the incidence of endophthalmitis after 23-gauge PPV, and to investigate the clinical settings, management strategies, causative organisms, and visual acuity outcomes for these cases.

## Methods

Data of the eyes that underwent 23-gauge PPV from January 2011 to December 2014 at the Eye Hospital of Wenzhou Medical University was retrospectively collected. All the information, including medical history, ocular examinations, and laboratory investigations, etc., was obtained from the electronic medical system. The exclusion criteria included: (1) pre-operative diagnosis of endophthalmitis; (2) history of vitrectomy; (3) intraocular surgery within 6 months; (4) history of ocular penetrating trauma; (5) sutures for any of the 3 sclerotomy incisions; (6) patients with cancer, acquired immune deficiency syndrome, or taking drugs that may influence the immune system. The diagnosis of endophthalmitis was based on ocular characteristics and culture results from operative sample. The clinical characteristics included: vision acuity reduction and eye pain, conjunctival congestion, hypopyon or fibrinous exudation, vitritis or vitreal empyema, and massive high-level echoes in the vitreous on the B-scan ocular ultrasonogram.

The aqueous and vitreous samples were collected at the beginning of the surgery, and consequent bacterial and fungal cultures were performed. This study followed the tenets of the Declaration of Helsinki and was approved by the Ethics Committee of the Eye Hospital of Wenzhou Medical University. Written, informed-consent of patients was obtained.

## Results

The data of 4593 eyes of Chinese patients underwent 23-gauge PPV from January 2011 to December 2014 was retrospectively reviewed. Six hundred fourteen eyes were excluded as per the exclusion criteria of this study. Hence, 3979 eyes from 6 surgeons were included for further analysis. Among which, 2122 eyes (2122/3979, 53.3%) received combined phacoemulsification and vitrectomy. Three eyes from two surgeons developed endophthalmitis postoperatively, giving the total incidence of 0.075% (3/3979). Among which, 2 eyes received the combined surgical procedure, giving the incidence of endophthalmitis 0.094% (2/2122) and 0.054% (1/1857) for eyes with combined surgery and single 23-gauge PPV, respectively (*p* = 1.0, Fisher exact test). The characteristics of the eyes without endophthalmitis were presented in Table 1.

**Table 1:** Characteristics of the eyes without endophthalmitis

	*N* = 3976
Age, years	56.0 ± 14.1
Gender (male/female)	2018/1959
Diabetes mellitus (n, %)	979, 24.6
Hypertension (n, %)	884, 22.2
Combined phacoemulsification (n, %)	2122, 53.4
Intravitreal use of TA (n, %)	124, 3.1
Tamponade (n, %)	
Fluid	930, 23.4
Air or gas	1347, 33.9
Silicone oil	1699, 42.7

*TA* triamcinolone acetonide

All the sclerotomy procedures were performed using the two-step technique. Phacoemulsification plus intraocular lens implant and C_2_F_6_ or air tamponade were also performed for the two elderly patients. Operative intraocular adjuvant (triamcinolone acetonide, TA) was used in 1 case. No postoperative subconjunctival antibiotic was used in either case. The detailed basic information on these 3 eyes with endophthalmitis is presented in Table 2.

**Table 2:** Detailed information of the three eyes developed endophthalmitis

	Case 1	Case 2	Case 3
Age, years	71	70	38
Gender	Male	Male	Male
Eye	Right	Right	Right
Primary ocular disease	VH, Terson syndrome	ERM	VH
Primary VA	0.05	0.3	0.2
Primary IOP, mmHg	15	10	17
Diabetes mellitus	Yes	No	Yes
Hypertension	Yes	No	Yes
Primary surgery	Phaco+IOL + PPV + ILM peeling+C_2_F_6_	Phaco+IOL + PPV + ERM peeling+air	PPV + ILM peeling+PRP + TA
Intravitreal use of TA	No	No	Yes
VA at day 1 post-operation	HM	0.3	0.4
IOP (mmHg) at day 1 post-operation	12	8	8

*VH* vitreous hemorrhage, *ERM* epiretinal membrane, *VA* visual acuity (Snellen), *IOP* intraocular pressure, *Phaco* phacoemulsification, *IOL* intraocular lens, *PPV* pars plana vitrectomy, *ILM* inner limiting membrane, *PRP* pan-retinal photocoagulation, *TA* triamcinolone acetonide

All cases received a vitreous tap, intravitreal injection with ceftazidime (20 mg/ml, 0.1 ml) and vancomycin (10 mg/ml, 0.1 ml). Silicone oil tamponade was performed in 2 eyes. All patients received systemic antibiotics (Table 3). The details of the 3 cases are reported below.

**Table 3:** Characteristics of the three eyes after developing endophthalmitis

	Case 1	Case 2	Case 3
Onset time, day	1	2	5
Symptom	VA reduction, eye pain	VA reduction, eye pain	VA reduction
Sign	hypopyon, fibrinous exudation in anterior chamber	conjunctival congestion, corneal edema, fibrinous exduation in anterior chamber	fibrinous exduation in anterior chamber, vitritis
VA before secondary surgery	LP	LP	HM
IOP (mmHg) before secondary surgery	15	19	14
Secondary surgery	ViT + PRP + SiO + ViAbI	ViT + PRP + SiO + ViAbI	lensectomy+ViT+ ViAbI
Systemic antibiotics	vancomycin, cephalothin	vancomycin, ceftazidime	Cefazolin
Culture result	enterococcus faecalis	Negative	*Staphylococcus epidermidis*
Follow-up time, month	73.6	32.2	10.5
VA at last visit	LP	0.05	0.5
IOP (mmHg) at last visit	8	9	11

*VA* visual acuity, *IOP* intraocular pressure, *HM* hand motions, *LP* light perception, *ViT* vitreous tap, *ViAbI* vitreous antibiotics injection, *SiO* silicone oil, *PRP* pan-retinal photocoagulation

### Case 1

A 71-year-old male had developed endophthalmitis just 1 day after PPV for vitreous hemorrhage. The patient felt visual acuity reduction and mild eye pain. There was moderate fibrinous exudation around the pupil, and the hypopyon accounted for about a quarter of the anterior chamber. A vitreous tap, panretinal photocoagulation, intravitreal antibiotics injection and silicone oil tamponade was performed at day 2 post-operation, after unsatisfactory intravitreal and systemic intravenous antibiotics injection. The culture results revealed enterococcus faecalis growth. At the last visit (73.6 months) without silicone oil removal, the patient’s visual acuity was not improved (light perception) because of macular necrosis.

### Case 2

A 70-year-old male had developed endophthalmitis 2 days after PPV for epiretinal membrane. The patient felt visual acuity reduction and moderate eye pain. There was moderate corneal edema and fibrinous exudation in the anterior chamber. A vitreous tap, intravitreal antibiotics injection, panretinal photocoagulation, and silicone oil tamponade was performed at day 4 post-operation, after unsatisfactory intravitreal and systemic intravenous antibiotics injection. The culture results were negative, showing nil growth. The silicone oil was removed 15.7 months later. At the last visit (32.2 months), the patient’s visual acuity was slightly improved from light perception to 0.05.

### Case 3

A 38-year-old male had developed endophthalmitis 5 days after PPV for vitreous hemorrhage. The patient felt visual acuity reduction, but without eye pain. There was moderate fibrinous exudation in the anterior chamber and apparent vitritis. An emergent lensectomy with vitreous tap and intravitreal antibiotics injection was performed. The culture results revealed growth of coagulase-negative *Staphylococcus epidermidis*. At the last visit (10 months), the patient’s visual acuity was improved from hand motions to 0.5.

## Discussion

In the current study, the incidence of endophthalmitis after 23-gauge PPV was found to be 0.075% (3 cases per 3979 eyes). This incidence was slightly higher than the previously reported incidence after 23-guage PPV, which ranged from 0 to 0.03% [8–10]. Scott et al. reported a stable rate of endophthalmitis after 20-gauge and 23 gauge PPV during 2007-2008, but a decreased rate after 25-gauge PPV, compared to that of 2005-2006 [9]. The authors speculated this decrease may be related to the increase of surgeons’ experience [9]. In a meta analysis from 6 large retrospective comparative cases series on the 25-gauge trans conjunctival sutureless vitrectomy (TSV) as compared to 20G PPV, Bahrani et al. concluded that 25G TSV does not have a higher rate of postoperative endophthalmitis compared to the 20G PPV [12]. Data from a 5-year Latin America multi-center retrospective study showed that the incidence of endophthalmitis was very similar (around 0.03%), after 20-gauge, 23-gauge and 25-gauge PPV [10]. A prospective, nationwide case-control study from the United Kingdom also showed that the small-gauge vitrectomy did not increase the risk of endophthalmitis [11]. Though they could not compare directly, the incidence after 23-gauge PPV was much lower than earlier reports on endophthalmitis after 25-gauge PPV, which was 0.84% [5]. This may further demonstrate that the small-gauge PPV per se was not related to the postoperative endophthalmitis.

The potential predisposing factors for endophthalmitis after small-gauge PPV includes immunosuppression, preoperative topical steroids [11], sutureless sclerotomy wounds, leaking sclerotomies causing early postoperative hypotony, patient-induced wound distortion (such as with eye rubbing), vitreous wick in the sclerotomies, increasing use of intravitreal adjuvants (such as TA, which potentially blunts the immune response to infection) [5], and straight sclerotomy incisions [5, 7, 9]. It was reported that angled incisions provide improved stability and watertight closure as compared to straight incisions [6, 7]. The incidence of endophthalmitis after straight incision was 0.18% to 0.23% for 25-guage PPV [6, 7], which was higher than angled incision 0 to 0.075% (current study) [7].

Combined intraocular surgery with PPV, such as phacoemulsification, penetrating keratoplasty, and glaucoma filtering surgery, has been touted to increase the risk of developing postoperative endophthalmitis. A 5-year multi-center retrospective data from Latin America showed that the incidence of endophthalmitis after small-gauge PPV without combined phacoemulsification was 0.028% and 0.021% for 23-gauge and 25-gauge, respectively [10]. Parolini et al. reported that none of 943 eyes after 23-gauge vitrectomy (38% combined with phacoemulsification and intraocular lens implantation) developed endophthalmitis [8]. Although Chen et al. reported that an apparent increase of the rate of endophthalmitis (2.17%, 1 of 46) after combining phacoemulsification cataract and 25-gauge vitrectomy compared to only 25-gauge vitrectomy (0.23%, 1 of 431), the sample size was too small to draw a conclusion [13]. Shimada et al. reported that the incidence of postoperative endophthalmitis was 0.0299% (1 of 3343 eyes) for 25-gauge vitrectomy [7]. The only case had combined phacoemulsification. However, it was unclear whether the 3343 eyes enrolled had combined surgical procedures [7]. Hence, with currently available retrospective studies, we do not have sufficient power to assess if combined cataract surgery and small gauge vitrectomy increases the risk of postoperative endophthalmitis. In this current study, the incidence of endophthalmitis was slightly higher (0.094%) for eyes with 23-gauge PPV and combined phacoemulsification surgery, compared to eyes with single 23-gauge PPV (0.054%). However, due to the very rare finding of endophthalmitis, it was not powerful enough to draw a statistical or clinical conclusion.

Subconjunctival antibiotics, conjunctival irrigation, adequate excision of peripheral vitreous, and air tamponade may also contribute towards preventing the onset of postoperative endophthalmitis [5, 7]. The air tamponade at the end of surgery may help to prevent postoperative fluid leakage, reduce the rate of postoperative hypotony, and limit the influx of bacteria [7, 12]. In a meta analysis, Bahrani et al. found that of the 22 patients who developed endophthalmitis, 19 patients did not undergo air-fluid exchange and the vitreous cavity remained fluid-filled at the end of the case [12]. In the current study, two of the three patients had diabetes mellitus for more than 10 years. Operative TA was used in one case, while postoperative gas tamponade was performed in one case, subconjunctival antibiotics and systemic antibiotics were used in none of the cases. Furthermore, silicone oil was tamponaded in none of the case. These may have accounted for the slightly higher incidence of endophthalmitis in this study.

Gram positive cocci were the most reported pathogenic organisms for endophthalmitis after PPV. In this study, 2 of 3 cases isolated coagulase-negative Staphylococci and Enterococcus, respectively. This is highly consistent with Scott et al.’s report that 86% of infections was caused by coagulase-negative Staphylococci, while the other 14% was caused by enterococcus [5]. Shimada et al. reported methicillin-resistant *S. aureus* and E. faecali were the causative bacteria after 20-gauge and 25-gauge PPV [7]. The culture organisms from 6 eyes with postvitrectomy endophthalmitis, from a 20-year retrospective study of the Bascom Palmer Eye Institute, showed *Staphylococcus aureus* (*n* = 3), Proteus mirabilis (*n* = 1), and *Staphylococcus epidermidis*/Pseudomonas aeruginosa (*n* = 1), while 1 case was negative [4]. A prospective, nationwide case-control study from the United Kingdom also showed that most of the endophthalmitis was caused by Staphylococci [14].

The visual acuity outcomes of this study varied from light perception to 0.5. The visual acuity outcomes after small-gauge PPV was also varied in the literature. Scott et al. reported an endophthalmitis after 23-gauge PPV infected by coagulase-negative Staphylococci, had limited final visual acuity of hand motions due to retinal detachment [9]. For endophthalmitis after 25-gauge PPV also infected by coagulase-negative Staphylococci and Enterococcus, had a much better visual acuity outcomes (20/400 to 20/25) [5]. Zhang et al. reported the final visual acuity ranged from light perception to 0.02 [3]. A two-decade study (1984-2003) from the Bascom Palmer Eye Institute reported that the final visual acuity of postvitrectomy endophthalmitis ranged from 20/200 to no light perception after treatment, with a third (4 out of 6 eyes) of light perception or worse [4].

In summary, this study reported the incidence of endophthalmitis after 23-gauge pars plana vitrectomy to be 0.075%, which was slightly higher than previous reports (around 0.03%). This sight-threatening postoperative complication had an acute onset, usually within 5 days. The most common pathogenic organism was gram positive cocci.

## Conclusions

Although rare, endophthalmitis is a potentially sight-threatening complication after 23-gauge pars plana vitrectomy. The infection, commonly caused by gram positive cocci, had its peak duration of onset, within 5 days post-operation.

## Additional file

Additional file 1: Dataset. (XLSX 11 kb)

## Abbreviations

PPV: pars plana vitrectomy
TA: triamcinolone acetonide
TSV: transconjunctival sutureless vitrectomy
